# Impacts of dioxin exposure on brain connectivity estimated by DTI analysis of MRI images in men residing in contaminated areas of Vietnam

**DOI:** 10.3389/fnins.2024.1344653

**Published:** 2024-04-25

**Authors:** Pham Ngoc Thao, Muneko Nishijo, Pham The Tai, Tran Ngoc Nghi, Takashi Yokawa, Vu Thi Hoa, Tran Viet Tien, Nguyen Xuan Kien, Tran Hai Anh, Yoshikazu Nishino, Hisao Nishijo

**Affiliations:** ^1^Department of Functional Diagnosis, Military Hospital 103, Vietnam Military Medical University, Ha Noi, Vietnam; ^2^Department of Epidemiology and Public Health, Kanazawa Medical University, Ishikawa, Japan; ^3^Institute of Biomedicine and Pharmacy, Vietnam Military Medical University, Ha Noi, Vietnam; ^4^Ministry of Health, Vietnamese Government, Hanoi, Vietnam; ^5^Kobe BMA Laboratory, BioView Inc., Kobe, Japan; ^6^Department of Infectious and Tropical Diseases, Military Hospital 103, Vietnam Military Medical University, Ha Noi, Vietnam; ^7^Department of Military Medical Command and Organization, Vietnam Military Medical University, Ha Noi, Vietnam; ^8^Department of Physiology, Vietnam Military Medical University, Ha Noi, Vietnam; ^9^Department of Sport and Health Sciences, Faculty of Human Sciences, University of East Asia, Yamaguchi, Japan

**Keywords:** dioxin, diffusion tensor imaging, fractional anisotropy, neurodevelopment, Vietnam

## Abstract

**Introduction:**

Effects of dioxin exposure on gray matter volume have been reported in previous studies, but a few studies reported effects of dioxin exposure on white matter structure. Therefore, this study was undertaken to investigate the impact of dioxin exposure on white matter microstructure in men living in the most severely dioxin-contaminated areas in Vietnam.

**Methods:**

In 2019 brain MRI scans from 28 men living near Bien Hoa airbase were obtained at Dong Nai General Hospital, Vietnam, on a 3 T scanner using a conventional diffusion tensor imaging sequence. Two exposure markers were indicated by perinatal exposure estimated by assessment of maternal residency in a dioxin-contaminated area during pregnancy and by measurement of blood dioxin levels. A general linear model was used to compare fractional anisotropy (FA) values in 11 white matter tracts in both hemispheres between groups with and without perinatal dioxin exposure and groups with high and low blood dioxin levels after adjusting for covariates.

**Results:**

The adjusted mean FA value in the left cingulum hippocampal part (CGH) was significantly lower in the perinatal dioxin exposure group compared with the group without perinatal dioxin exposure. The high blood TCDD group showed significantly reduced FA values in the left and right CGH and right uncinate fasciculus (UNC). Moreover, the high blood TEQ-PCDDs group showed significantly lower FA values in the left and right CGH and the left UNC. There were no significant differences in FA values between the groups with high and low TEQ-PCDFs levels or between the groups with high and low TEQ-PCDD/Fs levels.

**Discussion:**

It was concluded that dioxin exposure during the perinatal period and adulthood may alter the microstructure of white matter tracts in individuals with neurodevelopmental disorders.

## Introduction

In a 35-year follow-up study of 15 industrial workers in the Czech Republic occupationally exposed to high levels of 2,3,7,8-tetrachlorodibenzo-p-dioxin (TCDD), Urban et al. (2007) reported an increased prevalence of neuropsychological problems and a focal reduction of perfusion in various brain areas using single-photon emission computed tomography (Urban et al., 2007). In American veterans exposed to herbicides during the Vietnam war, the US government reported an increased prevalence of Parkinson’s disease (Board on Population Health and Public Health Practice, 2018). Martinez et al. (2021) followed up 316,000 veterans (98% men; mean age: 62 years) from 2001 to 2015, and reported that the prevalence of Alzheimer’s dementia was almost twice as high in exposed veterans than in unexposed veterans [Hazard ratio (HR): 1.68; 95% Confidence interval (CI): 1.59–1.77] (Martinez et al., 2021). Moreover, in an investigation of the impact of dioxin exposure on brain morphology using magnetic resonance imaging (MRI), Lee et al. (2022) showed significant brain atrophy progression in the bilateral frontal and temporal lobes in Korean veterans exposed to Agent Orange during the Vietnam war (Lee et al., 2022). Together, these observations suggest that exposure of the mature brain to dioxins from Agent Orange may cause changes in brain volume, particularly in areas related to cognitive functions.

We investigated the association between dioxin concentration in blood and gray matter volumes in fathers of children in the Bien Hoa birth cohort in our previous studies (Nghiem et al., 2019; Pham et al., 2021), and found that blood TCDD levels were associated with low gray matter volume in the left fusiform gyrus and the left medial temporal pole, while the toxic equivalent of polychlorinated dibenzo-p-dioxins (TEQ-PCDDs) was correlated with low medial temporal pole volume (Vu et al., 2021). We also found significantly reduced left inferior frontal gyrus pars orbitalis volume in men with perinatal exposure, estimated from maternal residency, compared with those without perinatal exposure (Vu et al., 2021). These results suggest that dioxin exposure during adulthood and perinatal dioxin exposure (i.e., during brain development) may alter the gray matter in various brain regions and have adverse neurological effects.

Changes in the white matter, including neuronal fibers, can be shown by diffusion tensor imaging (DTI), which provides information on the microstructural features of neural tracts (Le Bihan et al., 2001). Fractional anisotropy (FA) is commonly used as a MRI biomarker in DTI studies and reflects the directionality of diffusivity within a tract (Chanraud et al., 2010). Reduced FA values indicate a decrease in the connectivity of neuronal fibers, associated with alterations in myelination, axon diameter, axon density and membrane permeability (Chanraud et al., 2010; Jones et al., 2013), and is frequently found in neurodevelopmental disorders, such as attention deficit hyperactivity disorder (ADHD) (Damatac et al., 2022), and in neurodegenerative diseases, such as Parkinson’s disease (Rashidi et al., 2023).

In the present study, we investigated the effects of dioxin exposure in adulthood and the perinatal period on white matter structures, including neuronal tracts connecting brain regions, indicated by FA values in DTI analysis, in Vietnamese men with alterations in brain gray matter reported by Vu et al. (2021, 2023). Our findings should provide insight into the impact of long-term dioxin exposure on brain development, particularly because of the longer maturation period of the white matter (until about 40 years of age) (Hasan et al., 2007; Lebel et al., 2012), compared with the gray matter (until adolescence) (Giedd et al., 1999).

## Materials and methods

### Study subjects

**Figure 1:**
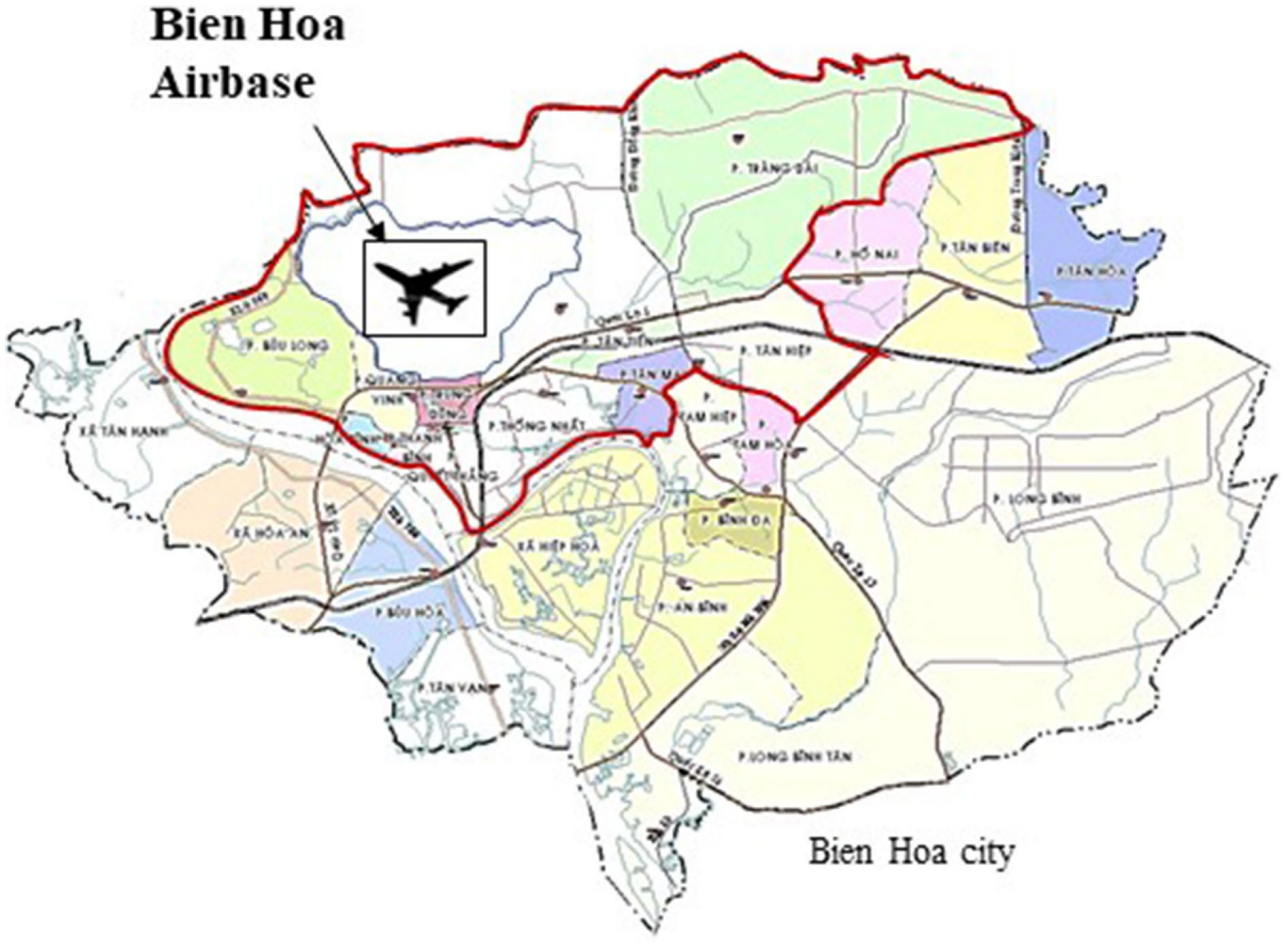
The map of the study.

A total of 78 mother-and-child pairs living in 10 communities near Bien Hoa airbase were recruited for this study (Figure 1). The mothers gave birth in Dong Nai prefectural hospital from August to December 2015 and the newborns were examined by electroencephalography (EEG) the day after birth. In 2018, we carried out a follow-up study at 2 years of age, with 61 children participating for general neurodevelopmental examination. At that time, we invited the fathers to join an investigation of blood dioxin concentration, and 40 agreed to participate. In 2019, these 40 fathers were invited to undergo brain MRI for the study, but only 33 (60%) participated, with seven men busy with work and absent on the examination day. Four men who showed left-handedness and one participant with extremely high blood TCDD levels (371.5 pg./g lipid) were excluded from further analysis. A total of 28 participants were included in the final analysis.

Information on age (years), education (years), smoking habit (yes), alcohol consumption (yes), length of residency in Bien Hoa City (yes), medical history (yes), job, working place related to the airbase (yes) or nearby industrial areas, the previous use of herbicides (yes) and the consumption of food grown on the airbase was recorded from the participant on the examination day. We also interviewed the mothers for their residency history, and found that 12 mothers lived near Bien Hoa airbase during pregnancy (1970 to 1992). Extremely high levels of TCDD in breast milk were previously reported in residents living near Bien Hoa airbase in samples collected in 1970–1973 and 1985–1988, and in blood samples collected in 1999 (Schecter et al., 2001). Furthermore, infant formula was not used as a common feeding method among residents of Bien Hoa City. We therefore surmised that the infants were fed breast milk before weaning. Vu et al. (2021, 2023) suggested that the mothers, who had lived in Bien Hoa before and after birth, were exposed to TCDD originating from Agent Orange even after spraying of the herbicide was discontinued.

The characteristics of the participants are displayed in Table 1. The average age and years of education were 35.8 and 11.6 years, respectively. The mean length of residency near Bien Hoa airbase was 19.3 years. Among the participants, 13 men (46.4%) were smokers, and 23 men (82.1%) consumed alcohol. However, only 3 men (10.7%) consumed alcohol daily. Furthermore, 3 men (10.7%) had a job inside the airbase or consumed meat grown on the airbase. Three men (10.7%) had a medical history, with two cases of hypertension and one case of gastritis. Among the men, 14 (50%) worked at nearby industrial areas, and 8 men (28.6%) previously used herbicides and pesticides for growing vegetables in their gardens. Their mean BMI was 24.4, with 13 men showing obesity (46.4%; BMI ≥ 25) (Table 1).

**Table 1:** The characteristics of the participants (*N* = 28).

Characteristics	Unit	Mean, *N*	SD, [%]
Age	Years	35.8	5.9
Education	Years	11.6	3.1
Smoking	Yes	13	[46.4]
Alcohol consumption	Yes	23	[82.1]
Length of residency	Years	19.3	14.7
Height	cm	165.3	5.2
Weight	kg	66.8	9.0
BMI	kg/m^2^	24.4	2.8
Medical history	Yes	3	[10.7]
Job related to the airbase	Yes	3	[10.7]
Worked nearby industrial areas	Yes	14	[50.0]
Used herbicides	Yes	8	[28.6]
Consumed food grown in the airbase	Yes	3	[10.7]
Their mother lived in Bien Hoa airbase during pregnancy	Yes	12	[42.9]
Blood dioxin concentration*			
TCDD	pg/g lipid	6.3	2.2
TEQ-PCDDs	pg-TEQ/g lipid	21.8	1.6
TEQ-PCDFs	pg-TEQ/g lipid	8.8	1.3
TEQ-PCDD/Fs	pg-TEQ/g lipid	31.2	1.4

N, Number of participants; SD, standard deviation; BMI, Body mass index; *geometrical mean and geometrical standard deviation; TCDD, 2,3,7,8- tetrachlorodibenzo-p-dioxin; TEQ, toxic equivalent; PCDDs, polychlorinated dibenzo-p-dioxins; PCDFs, polychlorinated dibenzo furans.

Written informed consent was obtained from all mothers according to a process reviewed and approved by the Health Departments of Bien Hoa City and Dong Nai Prefecture. The Institutional Ethics Board for medical and health research involving human subjects at Kanazawa Medical University (ES-187) and the University of Toyama (CS-26-30) approved the study design.

### MRI data acquisition and image processing

All subjects were scanned on a Siemens Magnetom Trio Tim system 3 T scanner using a conventional DTI sequence (Siemens, Erlangen, Germany) at the Department of Diagnostic Imaging in Dong Nai General Hospital, Vietnam. The parameters of the conventional DTI sequence were as follows: repetition time (TR) = 4,800 ms, echo time (TE) = 76 ms, slice thickness = 3.0 mm, 50 transverse slices without gap covering the whole brain, voxel size = 2 × 2 × 3 mm, field of view = 240 mm, 12 directions with *b* = 1,000 s/mm^2^ with an additional b0 (*b*-value = 0) image.

In the DTI data set, the *b* = 0 image was registered to MNI152_T2 using FLIRT and FNIRT (FSL 6.0.0). The other images of the DTI data set were spatially normalized by WARP (FSL) taking the coefficient-field made by the registration by FLIRT and FNIRT. Those normalized data were reconstructed by the Diffusion Toolkit into Diffusion-weighted Imaging, apparent diffusion coefficient, FA maps and TRK data for the visualization program TrackVis, which uses the fiber assignment by continuous tracking approach to reconstruct fiber paths. TrackVis can visualize and analyze fiber track data from diffusion MR imaging tractography. An angle threshold of 45° was selected to determine whether the fiber was in the same orientation. An angle higher than 45° indicated that the fiber was no longer part of the same fiber pathway. No FA threshold was set in TrackVis.

The white matter fiber tract orientation was displayed by yellow color. The DTI parameter, indicated by the FA value, which reflects the anisotropy or directionality of diffusion, was calculated.^1^

## Region of interest definition

**Figure 2:**
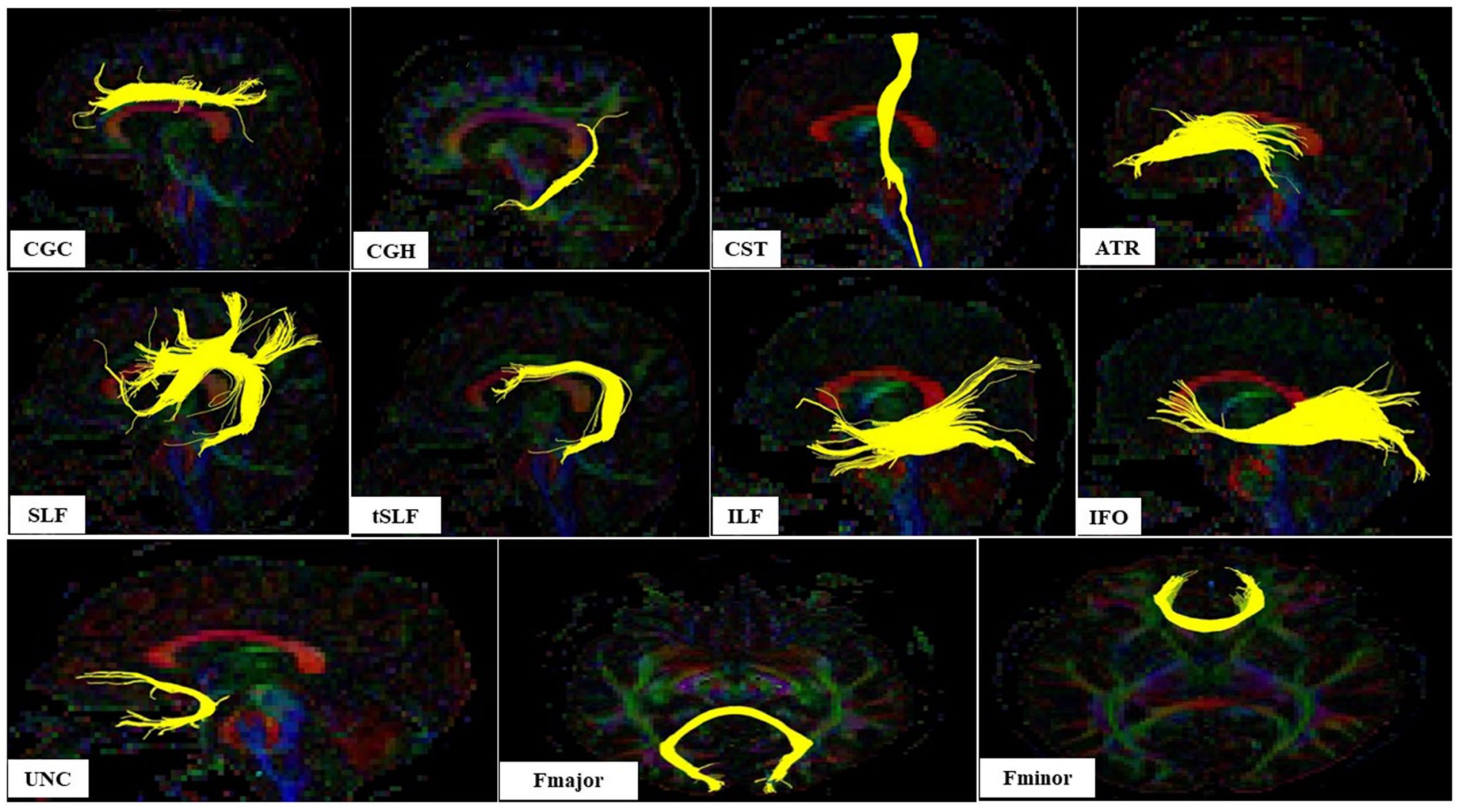
The sagital view of nine white matter tracts and axial view of two white matter tracts in left hemisphere evaluated according Wakana et al. (2007); CGC, Cingulum cingulate gyrus part; CGH, Cingulum hippocampal part; CST, Cortico-spinal tract; ATR, Anterior thalamic radiation; SLF, Superior longitudinal fasciculus; tSLF, The temporal component of the SLF; IFL, The inferior longitudinal fasciculus; IFO, The inferior fronto-occipital fasciculus; UNC, The uncinate fasciculus (UNC); Fmajor, The forceps major; Fminor, The frontal projection of the corpus callosum.

Regions of interest (ROIs) were manually created by a medical doctor blind to dioxin levels of the participants. The examiner was trained thoroughly by a specialist in radiology. A multi-ROI approach was used to reconstruct white matter fiber tracts of interest following the protocol in a previous report (Wakana et al., 2007). The fiber tracts of interest penetrated the manually defined ROIs, and tracking results were extracted. The analyzed tracts included the left and right cingulum cingulate gyrus part, the left and right cingulum hippocampal part (CGH), the left and right cortico-spinal tract, the left and right anterior thalamic radiation, the left and right superior longitudinal fasciculus (SLF), the left and right temporal component of the SLF, the left and right inferior longitudinal fasciculus, the left and right inferior fronto-occipital fasciculus, the left and right uncinate fasciculus (UNC), the forceps major, and the frontal projection of the corpus callosum (Figure 2).

### Dioxin exposure analysis

An approximately 20 mL volume of venous blood was collected at Bien Hoa Health Center in 2018. Blood samples were frozen and transferred to Japan on dry ice for quantification of 17 2,3,7,8-substituted PCDD and polychlorinated dibenzofuran (PCDF) congeners at Kanazawa Medical University, Uchinada, Japan.

After processing, whole blood samples were dehydrated using an EYELA freeze-dryer (FDU-1200, Tokyo-rika Inc., Tokyo, Japan). The fat content was determined using an ASE-200 accelerated solvent extractor (Dionex, Sunnyvale, CA, USA) before 13C-labeled 2,3,7,8-substituted PCDDs/Fs (DF-LCS-A40, Wellington Laboratories, Guelph, Canada) were added into samples as an internal standard. A multi-layered silica gel column was used to purify samples, and a single-layered column of activated carbon was employed to separate and collect the PCDD/Fs fraction. The final extracted solution was concentrated by nitrogen evaporators, and levels of 17 PCDD and PCDF congeners were measured on a gas chromatograph (HP-6980, Hewlett-Packard, Palo Alto, CA, United States) equipped with a high-resolution mass spectrometer (HR-GC/MS; MStation-JMS700, JEOL, Tokyo, Japan). If the compound fell below the detection limit, half of the limit of detection was recorded as the measured value. Levels of each congener was recorded as pg./g fat. The toxic equivalent (TEQ) of PCDD/Fs in each sample was calculated by summing up the values obtained by multiplying each congener concentration by its toxic equivalent factor referenced from the WHO 2005-TEF (Van den Berg et al., 2006). The details of the analysis methods are described elsewhere (Tawara et al., 2003; Van Luong et al., 2018). Geometrical means and standard deviations of TCDD and TEQ of PCDDs, PCDFs and PCDD/Fs in blood samples are shown in Table 1.

### Data analysis

SPSS version 21.0 (IBM, Armonk, NY, United States) was used for statistical analyses. Concentrations of 17 PCDD/F congeners and the TEQ values of PCDDs, PCDFs and PCDD/Fs in blood were logarithmically transformed (base 10) to improve normality. A general linear model was used to compare the FA values in 11 white matter tracts in each hemisphere, between the groups with and without perinatal dioxin exposure and the groups exposed to high and low dioxin levels, after adjusting for confounding factors; these were correlated with FA values (covariates), including age (years) and height (cm). The cut-off value for the high and low exposure groups was set at the 75th percentile value of blood TCDD, TEQ-PCDDs, PCDFs and PCDD/Fs concentrations. At this time, the cut-off values were 29.3, 10.4, and 39.5 pg-TEQ/g lipid for TEQ-PCDDs, PCDFs, and PCDD/Fs, respectively, and 10.8 pg./g lipid for TCDD.

At that time, the values was set to determine high and low exposure group for TCDD, TEQ-PCDDs, PCDFs and PCDD/Fs as.

## Results

### Comparison of the adjusted mean FA values between the groups with and without perinatal dioxin exposure

**Figure 3:**
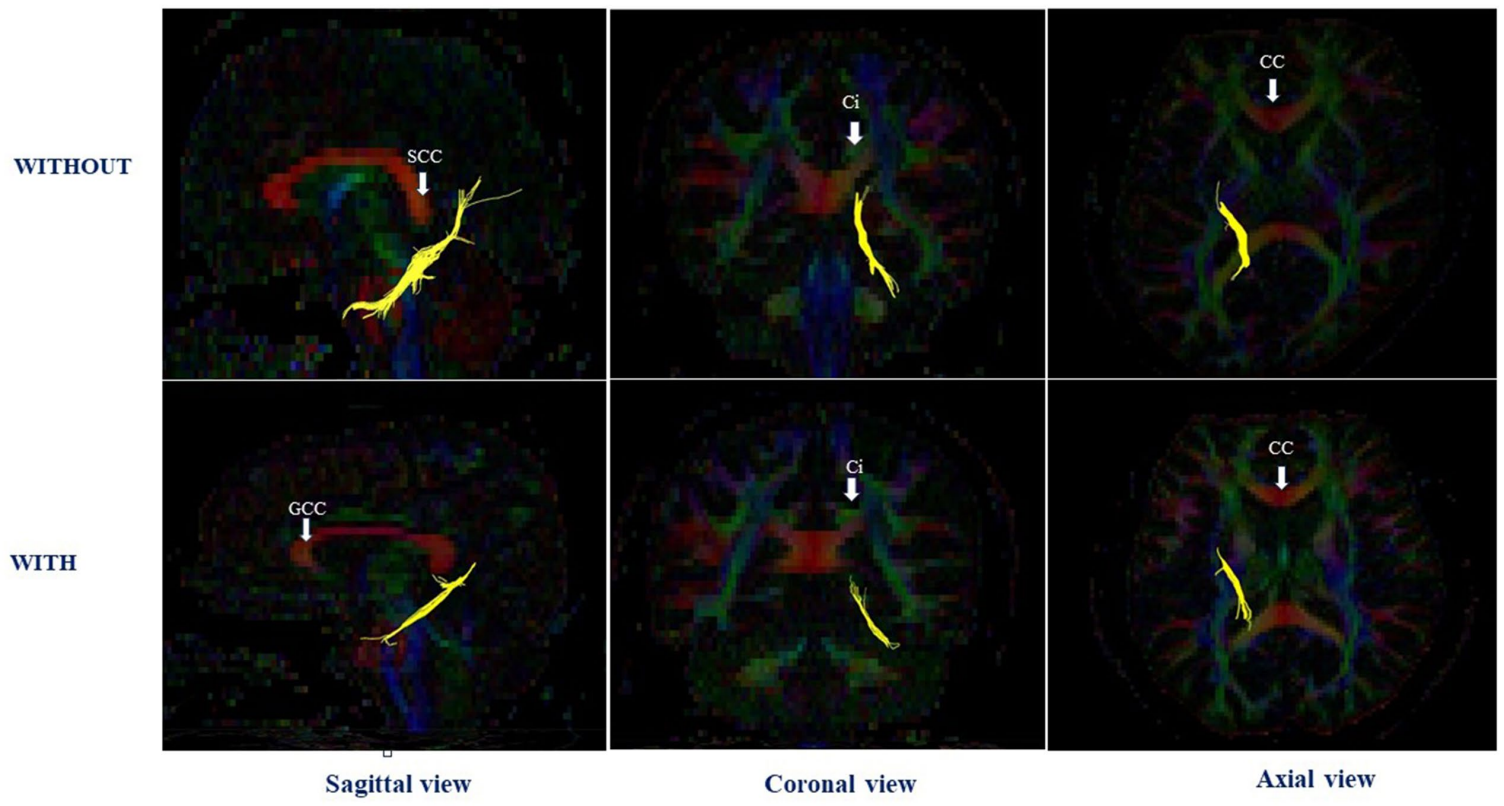
The left cingulum hippocampal part in case with and without perinatal dioxin exposure; SCC, the splenium of corpus callosum; CC, Corpus callosum; GCC, the genu of corpus callosum; Ci, Cingulum.

Table 2 shows the comparison of the adjusted mean FA values in white matter tracts between the groups with and without perinatal dioxin exposure. The FA value in the left CGH was significantly lower in the perinatal dioxin exposure group compared with the group without perinatal dioxin exposure (*p* < 0.05). The Figure 3 displayed the left CGH indicated by yellow color in case with and without perinatal dioxin exposure. There were no significant differences between these groups in FA values in other white matter tracts in the left hemisphere (*p* > 0.05). Similarly, no significant differences were observed between these two groups in FA values in white matter tracts in the right hemisphere (Table 2).

**Table 2:** Comparisons of the adjusted mean FA values between the groups with and without perinatal dioxin exposure.

	Perinatal dioxin exposure due to spraying of herbicides				
	Without (*N* = 16)		With (*N* = 12)		
FA values	Adj. mean	95% C.I. (lower, higher)	Adj. mean	95% C.I. (lower, higher)	*p*-value
**Left hemisphere**					
Cingulum cingulate gyrus part	0.537	(0.522, 0.552)	0.521	(0.503, 0.538)	0.169
Cingulum hippocampal part (CGH)	0.423	(0.414, 0.432)	0.409	(0.398, 0.419)	0.045
Cortico-spinal tract	0.584	(0.576, 0.593)	0.580	(0.570, 0.590)	0.495
Anterior thalamic radiation	0.447	(0.436, 0.457)	0.448	(0.435, 0.460)	0.895
Superior longitudinal fasciculus (SLF)	0.486	(0.475, 0.496)	0.482	(0.470, 0.495)	0.685
The temporal component of the SLF	0.515	(0.504, 0.527)	0.509	(0.495, 0.522)	0.441
Inferior longitudinal fasciculus	0.492	(0.481, 0.503)	0.496	(0.483, 0.509)	0.643
Inferior fronto-occipital fasciculus	0.524	(0.510, 0.538)	0.521	(0.505, 0.538)	0.778
Uncinate fasciculus (UNC)	0.442	(0.425, 0.459)	0.436	(0.416, 0.456)	0.617
The forceps major*	0.637	(0.625, 0.649)	0.641	(0.628, 0.655)	0.636
The frontal projection of the corpus callosum*	0.557	(0.545, 0.570)	0.565	(0.550, 0.580)	0.420
**Right hemisphere**					
Cingulum cingulate gyrus part	0.517	(0.505, 0.530)	0.506	(0.491, 0.520)	0.240
Cingulum hippocampal part (CGH)	0.418	(0.404, 0.433)	0.429	(0.412, 0.445)	0.337
Cortico-spinal tract	0.576	(0.565, 0.587)	0.576	(0.563, 0.588)	0.994
Anterior thalamic radiation	0.450	(0.435, 0.464)	0.440	(0.423, 0.457)	0.392
Superior longitudinal fasciculus (SLF)	0.482	(0.471, 0.492)	0.477	(0.466, 0.489)	0.584
The temporal component of the SLF	0.502	(0.490, 0.513)	0.497	(0.484, 0.510)	0.573
Inferior longitudinal fasciculus	0.495	(0.485, 0.506)	0.495	(0.483, 0.507)	0.996
Inferior fronto-occipital fasciculus	0.530	(0.519, 0.541)	0.520	(0.507, 0.533)	0.233
Uncinate fasciculus (UNC)	0.450	(0.439, 0.461)	0.444	(0.431, 0.457)	0.463

Covariates for: age, height. Adj. Mean, Adjusted mean; N, Number of subjects; FA, Fractional anisotropy; 95% C.I., 95% confidence interval; *: the white matter tract that connecting between two hemispheres.

### Comparison of the adjusted mean FA values between the high and low blood TCDD groups

The adjusted mean FA values in the white matter tracts in the right and left hemispheres were compared between the high and low blood TCDD groups after adjusting for confounding factors (Table 3). In the left hemisphere, the FA value in the CGH was significantly lower in the high TCDD group compared with the low TCDD group (*p* < 0.05). The FA value in the left cingulum cingulate gyrus part was lower in the high TCDD group compared with the low TCDD group, and this difference was nearly significant (*p* = 0.068). There were no significant differences between these groups in FA values in other white matter tracts.

**Table 3:** Comparisons of the adjusted mean FA values between the high and low blood TCDD groups.

	Low TCDD group (<10.8 pg./g lipid) (*N* = 21)		High TCDD group (≥10.8 pg./g lipid) (*N* = 7)		
FA values	Adj. mean	95% C.I. (lower, higher)	Adj. mean	95% C.I. (lower, higher)	*p*-value
**Left hemisphere**					
Cingulum cingulate gyrus part	0.536	(0.523, 0.549)	0.512	(0.489, 0.534)	0.068
Cingulum hippocampal part (CGH)	0.422	(0.415, 0.429)	0.400	(0.387, 0.412)	0.005
Cortico-spinal tract	0.585	(0.578, 0.593)	0.574	(0.561, 0.586)	0.119
Anterior thalamic radiation	0.449	(0.440, 0.459)	0.440	(0.423, 0.586)	0.320
Superior longitudinal fasciculus (SLF)	0.485	(0.475, 0.494)	0.483	(0.466, 0.500)	0.843
The temporal component of the SLF	0.512	(0.502, 0.522)	0.512	(0.495, 0.530)	0.988
Inferior longitudinal fasciculus	0.490	(0.485, 0.504)	0.490	(0.473, 0.507)	0.625
Inferior fronto-occipital fasciculus	0.524	(0.511, 0.536)	0.520	(0.511, 0.536)	0.797
Uncinate fasciculus (UNC)	0.443	(0.429, 0.458)	0.428	(0.402, 0.454)	0.306
The forceps major*	0.638	(0.628, 0.649)	0.641	(0.623, 0.660)	0.755
The frontal projection of the corpus callosum*	0.565	(0.554, 0.576)	0.550	(0.530, 0.569)	0.196
**Right hemisphere**					
Cingulum cingulate gyrus part	0.511	(0.500, 0.523)	0.515	(0.494, 0.535)	0.784
Cingulum hippocampal part (CGH)	0.418	(0.441, 0.418)	0.403	(0.383, 0.424)	0.033
Cortico-spinal tract	0.574	(0.565, 0.584)	0.579	(0.563, 0.596)	0.595
Anterior thalamic radiation	0.448	(0.435, 0.460)	0.440	(0.417, 0.463)	0.569
Superior longitudinal fasciculus (SLF)	0.480	(0.472, 0.489)	0.478	(0.462, 0.493)	0.768
The temporal component of the SLF	0.500	(0.491, 0.510)	0.498	(0.480, 0.515)	0.779
Inferior longitudinal fasciculus	0.496	(0.486, 0.505)	0.494	(0.478, 0.510)	0.905
Inferior fronto-occipital fasciculus	0.526	(0.516, 0.536)	0.526	(0.509, 0.544)	0.973
Uncinate fasciculus (UNC)	0.452	(0.443, 0.462)	0.432	(0.416, 0.448)	0.034

Covariates for: age, height. TCDD, 2,3,7,8- tetrachlorodibenzo-p-dioxin; Adj. Mean, Adjusted mean; N, Number of subjects; FA, Fractional anisotropy; 95% C.I., 95% confidence interval; *: only one track that connecting between two hemispheres.

**Figure 4:**
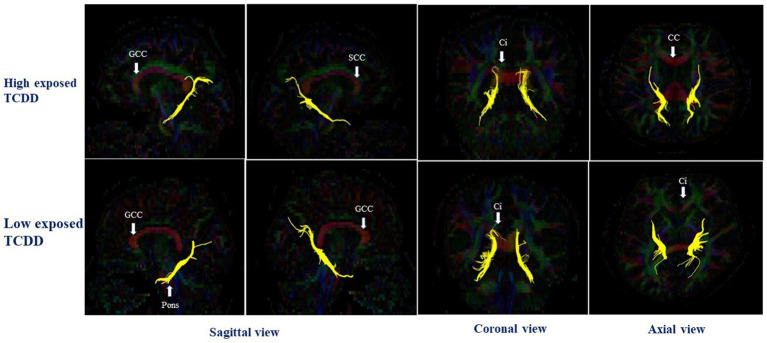
The left and right cingulum hippocampal part in case with high and low TCDD exposure; SCC, the splenium of corpus callosum; CC, Corpus callosum; GCC, the genu of corpus callosum; Ci, Cingulum.

**Figure 5:**
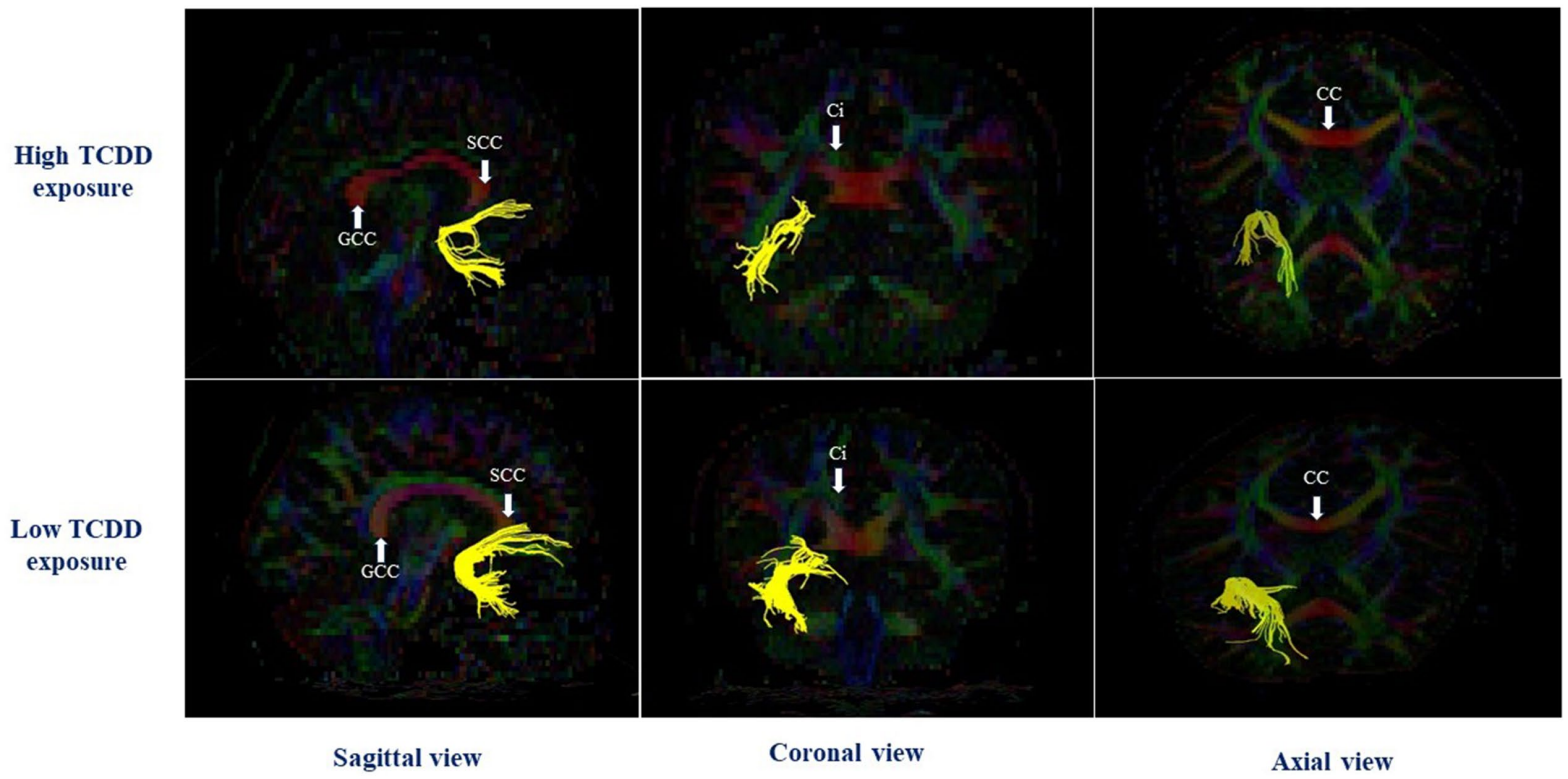
The right uncinate fasciculus in case with high and low TCDD exposure; SCC, the splenium of corpus callosum; CC, Corpus callosum; GCC, the genu of corpus callosum; Ci, Cingulum.

In the right hemisphere, the high TCDD group showed significantly decreased FA values in the CGH and UNC, compared with the low TCDD group (*p* < 0.05). No significant differences between these groups in FA values were found in other white matter tracts (Table 3). Also, the tracts of the left and right CGH, and right UNC in cases with high and low TCDD exposure was shown in Figures 4, 5.

### Comparison of the adjusted mean FA values between the high and low TEQ-PCDD/fs, TEQ-PCDDs, and TEQ-PCDFs groups

The adjusted mean FA values in 11 white matter tracts were compared between the groups with high and low TEQ-PCDD/Fs, TEQ-PCDDs and TEQ-PCDFs using a general linear model. There were no significant differences between the high and low TEQ-PCDD/Fs groups in FA values in the 11 white matter tracts (Table 4). Similarly, no significant differences between the high and low TEQ-PCDFs groups in FA values in white matter tracts were observed (unpublished data).

**Table 4:** Comparison of the adjusted mean FA values between the high and low TEQ-PCDD/Fs.

	Low TEQ-PCDD/Fs group (<39.5 pg-TEQ/g lipid) (*N* = 21)		High TEQ-PCDD/Fs group (≥39.5 pg-TEQ/g lipid) (*N* = 7)		
FA values	Adj. mean	95% C.I. (lower, higher)	Adj. mean	95% C.I. (lower, higher)	*p*-value
**Left hemisphere**					
Cingulum cingulate gyrus part	0.527	(0.514, 0.541)	0.538	(0.515, 0.562)	0.400
Cingulum hippocampal part (CGH)	0.419	(0.411, 0.427)	0.410	(0.396, 0.424)	0.281
Cortico-spinal tract	0.584	(0.577, 0.592)	0.577	(0.564, 0.590)	0.335
Anterior thalamic radiation	0.449	(0.439, 0.458)	0.442	(0.426, 0.459)	0.495
Superior longitudinal fasciculus (SLF)	0.484	(0.475, 0.494)	0.483	(0.467, 0.500)	0.907
The temporal component of the SLF	0.511	(0.501, 0.521)	0.516	(0.498, 0.533)	0.655
Inferior longitudinal fasciculus	0.493	(0.483, 0.503)	0.494	(0.478, 0.511)	0.883
Inferior fronto-occipital fasciculus	0.523	(0.511, 0.535)	0.522	(0.501, 0.544)	0.925
Uncinate fasciculus (UNC)	0.444	(0.430, 0.459)	0.425	(0.400, 0.450)	0.177
The forceps major*	0.638	(0.628, 0.649)	0.641	(0.623, 0.659)	0.816
The frontal projection of the corpus callosum *	0.560	(0.549, 0.571)	0.563	(0.543, 0.583)	0.811
**Right hemisphere**					
Cingulum cingulate gyrus part	0.511	(0.500, 0.523)	0.515	(0.495, 0.535)	0.735
Cingulum hippocampal part (CGH)	0.428	(0.416, 0.440)	0.407	(0.386, 0.428)	0.085
Cortico-spinal tract	0.572	(0.563, 0.582)	0.585	(0.569, 0.601)	0.166
Anterior thalamic radiation	0.446	(0.433, 0.459)	0.445	(0.423, 0.467)	0.944
Superior longitudinal fasciculus (SLF)	0.479	(0.470, 0.487)	0.483	(0.468, 0.499)	0.584
The temporal component of the SLF	0.498	(0.488, 0.508)	0.504	(0.487, 0.521)	0.508
Inferior longitudinal fasciculus	0.494	(0.485, 0.503)	0.498	(0.482, 0.513)	0.710
Inferior fronto-occipital fasciculus	0.525	(0.515, 0.535)	0.528	(0.511, 0.545)	0.748
Uncinate fasciculus (UNC)	0.448	(0.438, 0.458)	0.446	(0.429, 0.463)	0.850

Covariates for: age, height. Adj. Mean, Adjusted mean; N, Number of subjects; FA, anisotropy; 95% C.I., 95% confidence interval; TEQ-PCDD/Fs, toxic equivalent of polychlorinated dibenzo-p-dioxins and polychlorinated dibenzo furans; *: only one track that connecting between two hemispheres.

However, the high TEQ-PCDDs group showed significantly reduced adjusted mean FA values in the left and right CGH and left UNC compared with the low TEQ-PCDDs group (*p* < 0.05). There were no significant differences in FA values in other white matter tracts in the left or right hemisphere (Table 5).

**Table 5:** Comparisons of the adjusted mean FA values between the high and low TEQ-PCDDs group.

	Low TEQ-PCDDs group (<29.3 pg-TEQ/g lipid) (*N* = 21)		High TEQ-PCDDs group (≥29.3 pg-TEQ/g lipid) (*N* = 7)		
FA values	Adj. mean	95% CI (lower, higher)	Adj. mean	95% CI (lower, higher)	*p*-value
**Left hemisphere**					
Cingulum cingulate gyrus part	0.531	(0.517, 0.545)	0.528	(0.503, 0.552)	0.816
Cingulum hippocampal part (CGH)	0.421	(0.414, 0.429)	0.403	(0.390, 0.417)	0.027
Cortico-spinal tract	0.585	(0.578, 0.593)	0.574	(0.561, 0.587)	0.132
Anterior thalamic radiation	0.450	(0.441, 0.460)	0.437	(0.420, 0.453)	0.147
Superior longitudinal fasciculus (SLF)	0.486	(0.477, 0.495)	0.479	(0.462, 0.496)	0.468
The temporal component of the SLF	0.514	(0.504, 0.524)	0.509	(0.504, 0.524)	0.612
Inferior longitudinal fasciculus	0.495	(0.485, 0.505)	0.489	(0.472, 0.506)	0.537
Inferior fronto-occipital fasciculus	0.524	(0.512, 0.536)	0.520	(0.498, 0.542)	0.756
Uncinate fasciculus (UNC)	0.448	(0.434, 0.461)	0.416	(0.392, 0.439)	0.026
The forceps major*	0.639	(0.629, 0.650)	0.638	(0.620, 0.657)	0.908
The frontal projection of the corpus callosum*	0.564	(0.552, 0.575)	0.553	(0.533, 0.573)	0.356
**Right hemisphere**					
Cingulum cingulate gyrus part	0.512	(0.501, 0.524)	0.512	(0.491, 0.532)	0.945
Cingulum hippocampal part (CGH)	0.430	(0.419, 0.441)	0.400	(0.380, 0.419)	0.010
Cortico-spinal tract	0.575	(0.565, 0.585)	0.577	(0.560, 0.594)	0.799
Anterior thalamic radiation	0.448	(0.435, 0.461)	0.439	(0.417, 0462)	0.517
Superior longitudinal fasciculus (SLF)	0.481	(0.472, 0.490)	0.477	(0.461, 0.493)	0.668
The temporal component of the SLF	0.500	(0.490, 0.510)	0.498	(0.480, 0.515)	0.785
Inferior longitudinal fasciculus	0.496	(0.487, 0.505)	0.494	(0.478, 0.510)	0.850
Inferior fronto-occipital fasciculus	0.525	(0.515, 0.535)	0.528	(0.510, 0.545)	0.821
Uncinate fasciculus (UNC)	0.451	(0.441, 0.460)	0.437	(0.420, 0.453)	0.146

Covariates for: age, height. Adj. Mean, Adjusted mean; N, Number of subjects; FA, Fractional anisotropy; 95% C.I., 95% confidence interval; TEQ-PCDDs, toxic equivalency of polychlorinated dibenzo-p-dioxins; *: only one track that connecting between two hemispheres.

To investigate which PCDD congeners may have contributed to the FA value changes, we compared the adjusted mean FA values in the left and right CGH and left UNC with the PeCDD, HxCDD1, HxCDD2, HxCDD3, HpCDD and OCDD levels. The 75th percentile of the concentration value was used to divide subjects into the low and high exposure groups. However, no significant difference was found between the high and low exposure groups for any PeCDD, HxCDD1, HxCDD2, HxCDD3, HpCDD, or OCDD congener (data not shown).

## Discussion

### Dioxin exposure and neurodevelopmental disorders

The adjusted mean FA value in the left CGH was significantly lower in the perinatal dioxin exposure group compared with the unexposed group. The high blood TCDD group showed significantly reduced FA values in the left and right CGH and right UNC. Similarly, the high blood TEQ-PCDDs group showed significantly reduced FA values in the left and right CGH and left UNC. There were no significant differences in FA values between the groups with high and low TEQ-PCDFs levels or between the groups with high and low TEQ-PCDD/Fs levels. To the best of our knowledge, the present study is the first to report the effects of dioxin exposure on microstructural changes in white matter tracts, such as the CGH and UNC, which are involved in limbic system functions and which have been reported to be affected in individuals with neurodevelopmental disorders such as autism spectrum disorder (ASD) and ADHD.

In our previous epidemiological studies, we have reported the neurodevelopmental impacts of perinatal dioxin exposure indicated by dioxins in breast milk to increase ASD and/or ADHD in children from birth cohorts at various ages living in areas near former US airbases in Da Nang and Bien Hoa, Vietnam (Nishijo et al., 2014; Pham The et al., 2020; Pham et al., 2022; Pham-The et al., 2022; Thao et al., 2023). Increased autistic traits indicated by higher Autism Spectrum Rating Scale (ASRS) scores and associated with higher perinatal TCDD exposure were found in 3-year-old children from Da Nang birth cohort recruited in 2008–9, particularly in boys (Nishijo et al., 2014). At 8 years of age, significantly increased ADHD behavior, particularly hyperactivity/impulsivity, were observed in Da Nang girls exposed to higher levels of TCDD during perinatal period (Pham-The et al., 2022).

In the areas around Bien Hoa airbase, 3-year-old girls from a Bien Hoa birth cohort recruited in 2012 showed atypical gaze behavior indicated by lower fixation density on faces (face fixation duration) in statistic pictures associated with perinatal TCDD exposure, which was inversely correlated with the scores of social communication scale, one of ASRS subscales (Pham et al., 2022). Gaze behavior was also investigated in 2-year-old children in Bien Hoa recruited in 2015 when viewing dynamic social stimuli. Reduced face fixation duration was found in boys in higher TCDD exposure group compared with lower exposure group (Thao et al., 2023). When they reached to 3 years of age, higher ASRS scores indicating increased autistic traits were found in higher perinatal TCDD exposure group in both sexes (Thao et al., 2023). Moreover, in the fathers of Bien Hoa children recruited in 2015, who participants in the current study, we reported significantly increased social anxiety symptoms associated with perinatal dioxin exposure (Vu et al., 2023), suggesting that our subjects may have social cognitive deficits similar to ASD associated with perinatal exposure to dioxins including high levels of TCDD originating from Agent Orange from Bien Hoa airbase.

In the clinical DTI studies, reduced FA values in the CGH in both hemispheres were reported in individuals with ASD (Bubb et al., 2018), suggesting that alteration of CGH connectivity is a frequent feature of ASD. In adolescents with ADHD, symptom severity was associated with FA values in the left CGH; however, higher FA values (hyperconnectivity) were associated with increased severity of ADHD symptoms (Cooper et al., 2015). Another DTI study reported lower FA values (hypoconnectivity) correlated with attention deficit in individuals with ADHD (Chiang et al., 2016). Konrad and Eickhoff (2010) suggested that either hyperconnectivity or hypoconnectivity may be observed in patients with ADHD (Konrad and Eickhoff, 2010).

Taken together, lower FA values in the CGH found in men with perinatal dioxin exposure in the current study may be associated with increased ASD or ADHD traits due to TCDD exposure during perinatal period. In addition, we found that FA values in the left and right CGH were lower in men with high TCDD and TEQ-PCDDs in blood. These results suggest that TCDD exposure during adulthood may also alter the microstructure of the CGH of adult brain, which are similar to those observed in patients with ASD and ADHD.

In the current study, we also found that men with higher blood TCDD levels showed lower FA values in the right UNC. Lower FA value in the left UNC associated with higher blood TEQ-PCDDs was also observed among them. A previous clinical DTI study in individuals with ASD reported significant asymmetrical changes in FA values, particularly lower FA values in the left UNC; however, their results varied greatly in the association between autism and changes in the UNC, including increased or decreased FA values, as well as in the handedness of their subjects (Olson et al., 2015). Moreover, some clinical studies reported differences in the volume, length, shape, and density of the UNC in individuals with ASD. Some studies showed greater UNC volume in the left hemisphere compared with the right hemisphere in individuals with ASD (Pugliese et al., 2009; Thomas et al., 2011), suggesting that the UNC is a white matter tract strongly associated with ASD. Notably, the UNC continues to develop long past adolescence, reaching peak maturity around 30 years of age (Lebel et al., 2012). These findings in clinical studies suggest that the UNC is at greater risk of being influenced by dioxin exposure in adulthood. Thus, exposure during adulthood to PCDD congeners, including TCDD, might cause microstructural changes in the UNC and decrease connectivity indicated by FA values observed in men of the current study.

Gray matter abnormalities related to social and behavioral deficits in patients with autism are often observed in various frontal and temporal gyri, including the superior and middle temporal gyri, and in motor and executive areas of the frontal lobe, which are considered components of the autism-specific structural network (Grecucci et al., 2016). Previously, we performed MRI analysis using voxel-based morphometry (VBM) and reported that perinatal dioxin exposure was associated with increased gray matter volume of the gyri in the autism-specific structural network in the same subjects as in the present study (Vu et al., 2023). Significant enlargement of the temporal pole, including the anterior region of the superior temporal gyrus, which plays an important role in social behaviors (Zahn et al., 2007), was also detected in men with perinatal exposure (Vu et al., 2023). Whereas, reduced gray matter volume in the left inferior frontal gyrus pars orbitalis, associated with perinatal dioxin exposure, and decreased gray matter volume in the left fusiform gyrus and left medial temporal pole, as well as lower medial temporal pole volume, associated with blood dioxin levels, were found in the same men by the statistical analysis using the cluster-based false-discovery rate (FDR) for multiple comparisons (SPM12 software package) (Vu et al., 2021). Taken together, these findings suggest that dioxin exposure, both perinatally and in adulthood, may influence both gray matter and white matter in the frontal and temporal lobes, which are involved in social and emotional behavior.

### Dioxin exposure and mild cognitive impairment and Alzheimer’s disease

Martinez et al. (2021) reported a nearly 2-fold higher prevalence of dementia in veterans exposed to Agent Orange (Martinez et al., 2021). Because the CGH plays important roles in memory and cognition (Ezzati et al., 2016; Bubb et al., 2018), white matter microstructural changes (reduced FA) in the posterior cingulate and CGH have been investigated in many studies on individuals with mild cognitive impairment (MCI) or Alzheimer’s disease (Bubb et al., 2018). The UNC plays important roles in episodic memory, language, and social emotional processing (Von Der Heide et al., 2013; Olson et al., 2015). Several DTI studies have compared FA values between individuals with MCI and healthy controls, and lower FA values were observed in the left or bilateral UNC in the former (Olson et al., 2015). These results suggest that reduced FA values in the CGH and UNC, associated with dioxin exposure in the current study, may contribute to the increased prevalence of MCI and Alzheimer’s disease in exposed men. Further longitudinal studies with a larger number of subjects, including elderly men, are needed to more fully elucidate the effects of dioxin exposure on the white matter, particularly the CGH and UNC, using DTI, as well as VBM analysis of gray matter regions.

### Possible mechanism of the effect of dioxin exposure on the white matter

Decreased FA values in the white matter are associated with demyelination, alterations of axon diameter and density, and changes in membrane permeability (Chanraud et al., 2010; Jones et al., 2013). Dioxins, particularly TCDD is thought to exert its biological and toxicological effects primarily by binding to the aryl hydrocarbon receptor (AhR), which is a ligand-activated transcription factor that mediate the expression of a diverse set of genes through the dioxin-responsive elements in the promoter regions of target genes (Beischlag et al., 2008). Neurons in the brain mainly of the cortex, the cerebellum, the hippocampus, the olfactory bulb, the hypothalamus, and the pituitary gland are suggested as cellular targets (Juricek and Coumoul, 2018) and susceptible to modulations in AhR activity, particularly in early developmental stages (Martin et al., 2022).

The AhR, however, expressed not only in the neurons but also in glia cells such as astrocytes and microglial cells, suggesting TCDD may affect these cells in white matter of the brain as a AhR ligand. In animal studies, TCDD exposure has been reported to reduce axonal growth (Iida et al., 2013), to disrupt dendritic growth in various areas of the brain, particularly the hippocampus and amygdala (Kimura et al., 2015), and to affect glial cell density in the corpus callosum (Reyes-Haro et al., 2013). *In vitro* studies, TCDD treatment disrupted communication between astrocytes and neurons of rat hippocampal culture (Legare et al., 2000), suggesting that TCDD may disturb glial functions to help formation and maintenance of synapses (Pfrieger, 2010) and to control of dendrite shape (Procko and Shaham, 2010). In addition, TCDD was shown to inhibit astrocytic differentiation of C6 glioma cells (Takanaga et al., 2004) and to stimulate proliferation of HAPI microglial cells derived from primary rat microglia-enriched cultures (Xu et al., 2014).

TCDD exposure has also been associated with destructive and inflammatory changes triggered by the AhR and stimulated glia cells to increase inflammation cytokines (Mitsui et al., 2011), resulting demyelination in the hippocampus (Rosińczuk et al., 2015) and delayed developmental myelination in several brain regions (Fernández et al., 2010).

Taken together, TCDD exposure may impact on not only neurons in gray matter of brain but also glia cells in white matter which have important roles in myelination, formation and maintenance of synapses, and axonal and dendritic growth. These changes would in turn disrupt neurotransmission and compromise white matter connectivity among brain areas, particularly those involving the limbic system.

### Limitations

The present study has some limitations, including the small number of subjects, which prevented us from analyzing the relationships between blood dioxin exposure, clinical symptoms and FA values. Another limitation is the lack of a control group from herbicide unexposed regions. At the beginning of the study, an MRI survey was also planned for fathers of children in a control area in northern Vietnam. However, we could not find a hospital with an MRI scanner using conventional DTI sequence in Hanoi, where our unexposed birth cohort is followed. In a future study, we will conduct an MRI survey in an unsprayed area other than Hanoi and reanalyze data to compare brain regional volumes in Bien Hoa fathers and controls.

Although gender-specific effects of dioxin have been observed in previous studies (Pham et al., 2019, 2022), only men were recruited in the present study. Therefore, future studies should include women to uncover any gender-associated differences in the effects of dioxins on the brain.

## Conclusion

Estimate perinatal dioxin exposure was associated with decreased FA values in the left CGH. High dioxin exposure during adulthood, indicated by high blood dioxin levels, were associated with decreased FA values in the CGH in both hemispheres, as well as the right or left UNC. Collectively, our findings suggest that dioxin exposure during the perinatal period and/or adulthood may cause microstructural changes in white matter tracts that often show altered connectivity in individuals with neurodevelopmental disorders.

## Data availability statement

The raw data supporting the conclusions of this article will be made available by the authors, without undue reservation.

## Ethics statement

The studies involving humans were approved by The Institutional Ethics Board for medical and health research involving human subjects at Kanazawa Medical University (ES-187) and the University of Toyama (CS-26-30) approved the study design. The studies were conducted in accordance with the local legislation and institutional requirements. The participants provided their written informed consent to participate in this study.

## Author contributions

PNT: Conceptualization, Data curation, Formal analysis, Investigation, Methodology, Software, Validation, Writing – original draft, Writing – review & editing. MN: Conceptualization, Data curation, Funding acquisition, Investigation, Methodology, Project administration, Resources, Supervision, Visualization, Writing – review & editing, Writing – original draft. PTT: Investigation, Data curation, Writing – original draft. TY: Data curation, Formal analysis, Software, Writing – original draft. TN: Data curation, Investigation, Writing – original draft. VH: Investigation, Data curation, Validation, Writing – original draft. TT: Conceptualization, Data curation, Writing – original draft. NK: Conceptualization, Methodology, Writing – original draft. TA: Investigation, Methodology, Writing – original draft. YN: Conceptualization, Methodology, Supervision, Project administration, Writing – original draft, Writing – review & editing. HN: Conceptualization, Methodology, Resources, Supervision, Visualization, Writing – review & editing, Formal analysis, Software, Project administration.

## Footnotes

^1^https://mriquestions.com/dti-tensor-imaging.html
